# In their own words: older persons’ experiences of participating in co-creation

**DOI:** 10.1186/s40900-025-00725-z

**Published:** 2025-05-26

**Authors:** Annikki Arola, Marlene Sandlund, Magdalena Eriksson Domellöf, Morag E. Taylor, Annika Toots

**Affiliations:** 1 Department of Community Medicine and Rehabilitation, Umeå University, Umeå, Sweden; 2 Department of Psychology, Umeå University, Umeå, Sweden; 3 School of Health Sciences, Faculty of Medicine and Health, UNSW Sydney, Sydney, Australia; 4 Graduate School and Research, Arcada UAS, Helsinki, Finland; 5 Falls, Balance and Injury Research Centre, Neuroscience Research Australia, Sydney, Australia

**Keywords:** Co-creation, Fall prevention, Older persons, Subjective experience

## Abstract

**Background:**

Co-creation methods ensure that interventions are tailored to the target group by incorporating their unique insights and preferences, strengthen innovation, and facilitate implementation. Although co-creation research is becoming more common, most research exploring co-creation focuses on the researchers’ perspectives rather than the experiences of the target population. By exploring these experiences, researchers can better understand the preferences for, and facilitators/barriers to, engagement and participation to inform future co-creation studies. This study aimed to explore older persons’ experiences and insights into participating in co-creation of an intervention to prevent falls.

**Methods:**

Qualitative interviews were conducted with 13 community-dwelling older persons (aged 66–83 years) after their participation in a co-creation study developing an intervention for fall prevention. Data were analyzed using qualitative content analysis.

**Results:**

Three themes emerged from the analysis: Diversity of co-creators enriches understanding and creativity, Interactive activities promote learning, and Supportive environments enhance collaboration. These themes describe how participating in workshops with others deepened and broadened participants’ knowledge and understanding of the subject and enabled them to contribute their experiences and perspectives. Discussing and testing exercises gave participants new insights into their physical abilities and the importance of exercise and a better understanding of the concept of motor-cognitive exercises and their role in everyday life. A respectful atmosphere where everyone shared responsibility for creating a supportive environment so all participants could express their thoughts was perceived as important by the participants.

**Conclusions:**

The results underscore the potential for co-creation to enhance participants’ knowledge and understanding of the topic, as well as their own capacity. For researchers, it is important to consider how to foster an inclusive and supportive environment, thereby boosting participation, engagement and collaboration.

**Supplementary Information:**

The online version contains supplementary material available at 10.1186/s40900-025-00725-z.

## Abstract (plain language summary)

The purpose of co-creation research is to engage end-users in developing relevant, feasible, and suitable interventions for implementation. While more studies are exploring the experience of participating in co-creation, they often focus on the researchers’ perspectives. It is important to explore the end-users’ experiences to understand their preferences for engagement and participation, as well as barriers and facilitators to engagement and participation. A better understanding of these factors will help future co-creation projects to better meet the end-users’ needs. We interviewed older persons who participated in a co-creation study to develop a fall prevention exercise program. This included taking part in workshops that involved discussing and practicing exercise with their peers, and homework tasks. After the workshops, we conducted individual interviews with 13 participants, aged 66–83 years, and analyzed the data using qualitative content analysis. The results suggested that participants gained new knowledge and insights into their fall risk and the importance of exercise to maintain and improve balance. They enjoyed sharing their knowledge and experiences. The co-creation process also provided them with a deeper understanding of their own abilities. Trust and inclusion in group interactions were crucial, enabling everyone to share ideas. The interactive activities during the workshops enhanced the learning experience. In conclusion, co-creation proved to be a valuable and enriching experience for older persons, fostering personal growth and practical application of knowledge. Combining hands-on activities and interactive engagement strengthened their understanding and confidence, while group dynamics played a crucial role in sustaining motivation and participation. These findings highlight the importance of inclusive and interactive approaches in collaborative initiatives involving older adults.

**Supplementary Information:**

The online version contains supplementary material available at 10.1186/s40900-025-00725-z.

## Background

To promote active and healthy aging, it is essential to develop interventions that support older persons to maintain their independence and health in everyday life [1]. Co-creation has been proposed as a suitable method to develop feasible and appropriate interventions for older adults and Leask et al. define co-creation within public health as “collaborative public health intervention development by academics working alongside other stakeholders” [2]. Co-creation in the healthcare sector focuses on the collaborative processes between healthcare providers and stakeholders to improve healthcare services. In co-creation the focus can be on different aspects, for example, that participants give their opinions on a specific issue or process or that they are actively involved in formulating research questions [3]. Co-creation enhances the quality of services, which can lead to more efficient and accessible healthcare [4]. Research also indicates that co-creation may have a positive impact on health, wellbeing and quality of life for health care users [5]. The collaborative approach in co-creation fosters a sense of inclusion and belonging, making the research process more meaningful for all involved [6].

A major threat to healthy aging is accidental falls [1]. Injuries caused by falls can be serious and negatively impact the individual’s well-being [7], abilities to function, and independence in daily life [8]. Evidence suggests that balance and strength exercises can reduce the risk and frequency of falls [9]. Moderate to high-intensity, progressive exercise programs have been shown to be most effective for fall prevention [10, 11]. Given the link between cognition and balance and walking ability, there may be a rationale for training both simultaneously, known as dual-task or motor-cognitive training [12]. Research indicates that motor-cognitive interventions can effectively improve balance and reduce the fear and risk of falls [11]. Despite strong evidence for exercise to prevent falls, implementation and long-term adherence remains a challenge [13].

Co-creation of interventions may help to overcome low adherence rates [1, 14]. A co-creation approach can increase the engagement of the target population and strengthen innovation and opportunities for implementation. Doing so can lead to more relevant and solutions-based research [15–17] and strengthen the credibility of the results [17]. There has been an increase in studies adopting a co-creation approach. According to Cowdell et al., the scientific literature related to the experience of co-creation typically offers an external perspective, mostly based on the researchers’ views [18]. End-user experiences have not been well explored [19]. Capturing participants’ experiences in co-creation will help researchers understand the participants’ preferences for engagement and participation, as well as the barriers and facilitators to participation and engagement, which may help inform future co-creation projects. Therefore, this study aimed to explore older persons’ experiences and insights when participating in the co-creation of an intervention to prevent falls.

## Methods

### Study context

This qualitative study describes older participants’ experiences of participating in co-creation involving a series of workshops to develop a motor-cognitive exercise intervention to prevent falls. The workshops followed the co-creation approach, with an iterative structure involving planning, conducting, reflecting, and evaluating [2]. Interactive activities in the workshops included brainstorming ideas and solutions, reflection, discussion of scenarios and testing early examples or prototypes of exercises. In total, six workshops were conducted over ten months, each workshop lasted approximately 2.5 h. The first five workshops were conducted in Northern Sweden during February to April 2023, and the final workshop in December 2023.

The first workshop introduced participants to the project, the co-creation process, falls prevention, and the motor-cognitive concept. In the second workshop, participants tested and discussed motor-cognitive exercises. The third workshop involved sharing experiences and testing motor-cognitive exercises with physiotherapists. The fourth workshop focused on brainstorming and testing different modes for delivery and packaging. In the fifth workshop, participants discussed scenarios for the wider application of motor-cognitive exercises. Finally, the sixth workshop involved testing and discussing prototypes of motor-cognitive exercises.

The workshops included 17 community-dwelling participants aged 65 years or older who were able to: 1) rise from a chair without personal assistance, 2) travel to the university campus, either independently or with assistance, and 3) communicate in group situations. Exclusion criteria were: moderate or severe dementia, palliative care, and living in a nursing home. In addition, the co-creation included four physiotherapists, actively working in geriatric rehabilitation, who took part in selected workshops but not in the qualitative interviews used for this study. Four of the authors (AA, MS, MED, AT) participated as researchers and also acted as moderators in the workshops. The participants were given written and verbal information about the study, including the qualitative interviews, and informed that participation was voluntary. All participants gave written consent to participate in the study. The Swedish Ethical Review Authority approved the study (Dnr 2022-05826-01).

### Participants

**Table 1:** **Table 1: Demographic characteristics of the participants**

Characteristics	Older adults (*n* = 13)
Age, years, mean (range)	74 (66–83)
Women, n (%)	4 (30.7)
Lives alone, n (%)	4 (30.7)
Fallen previous year, n (%)	7 (53.8)
Tertiary educational level n (%)^*^	6 (46.1)

*Based on International Standard Classification of Education 2011 [20]

At the fifth workshop, the older participants were reminded of the planned interviews to explore their experiences and insights into participating in the co-creation process and were invited to be interviewed. Table 1 presents the demographic information about the group. Thirteen (76%) of the 17 older participants agreed to a one-on-one interview and provided verbal informed consent (written consent was obtained at study outset). Four women and nine men, with a mean age of 74 years (range: 66–83 years) participated. 31% (*n* = 4) lived alone while nine persons (69%) lived together with someone else. Seven (54%) participants had experienced at least one fall during the last year. Their educational level was divided into tertiary education (*n* = 6) and secondary education (*n* = 7) according to International Standard Classification of Education [20]. All were independent in their daily activities and lived independently in the community.

### Data collection

All interviews were conducted by the first author (AA) at the participant’s home or on the university campus, depending on the participant’s preference. The interviews were conducted one month after the fifth workshop, once with each participant. An interview guide, informed by the Participatory and Appreciative Action and Reflection process (PAAR) [2], was used for the semi-structured interviews. Questions related to the co-creation process such as reframing, ownership, and participation [2]. The questions focused on participants’ experiences and insights during co-creation, including group cooperation and their own contributions, insights, and reflections. Follow-up questions were used to further explore participants’ experiences, as needed. Interviews of 25–45 min duration were audio recorded and transcribed verbatim by a certified communications company.

### Data analysis

Qualitative content analysis [21] was used to analyze the data using MAXQDA2022 [22] for data organization and management. The first author listened to and read all the interviews several times. The remaining authors (MS, MED, MET, AT) each read a selection of the interviews to familiarise themselves with the data. In the next step, the first author reread the transcriptions and divided the text into meaning units labeled with a code that described the content. The codes were discussed in the author group and grouped into subcategories based on similarities. Subsequently, the first author analyzed the subcategories by their content and compared them to form categories based on their similarities and differences. Through continuous discussions with co-authors, the categories were iteratively revised until consensus was reached regarding the final categories. The research group represented professionals in rehabilitation and psychology with diverse scientific expertise and experience in research approaches such as qualitative research and co-creation and expertise in fall prevention, exercise, cognitive training and motor-cognitive training. Additionally, all the authors had pedagogic competencies. The reporting adheres to the Short Form checklist outlined in the Guidance for reporting patient and public involvement (GRIPP2) [23]. (Supplementary file 1).

## Results

**Fig. 1:**
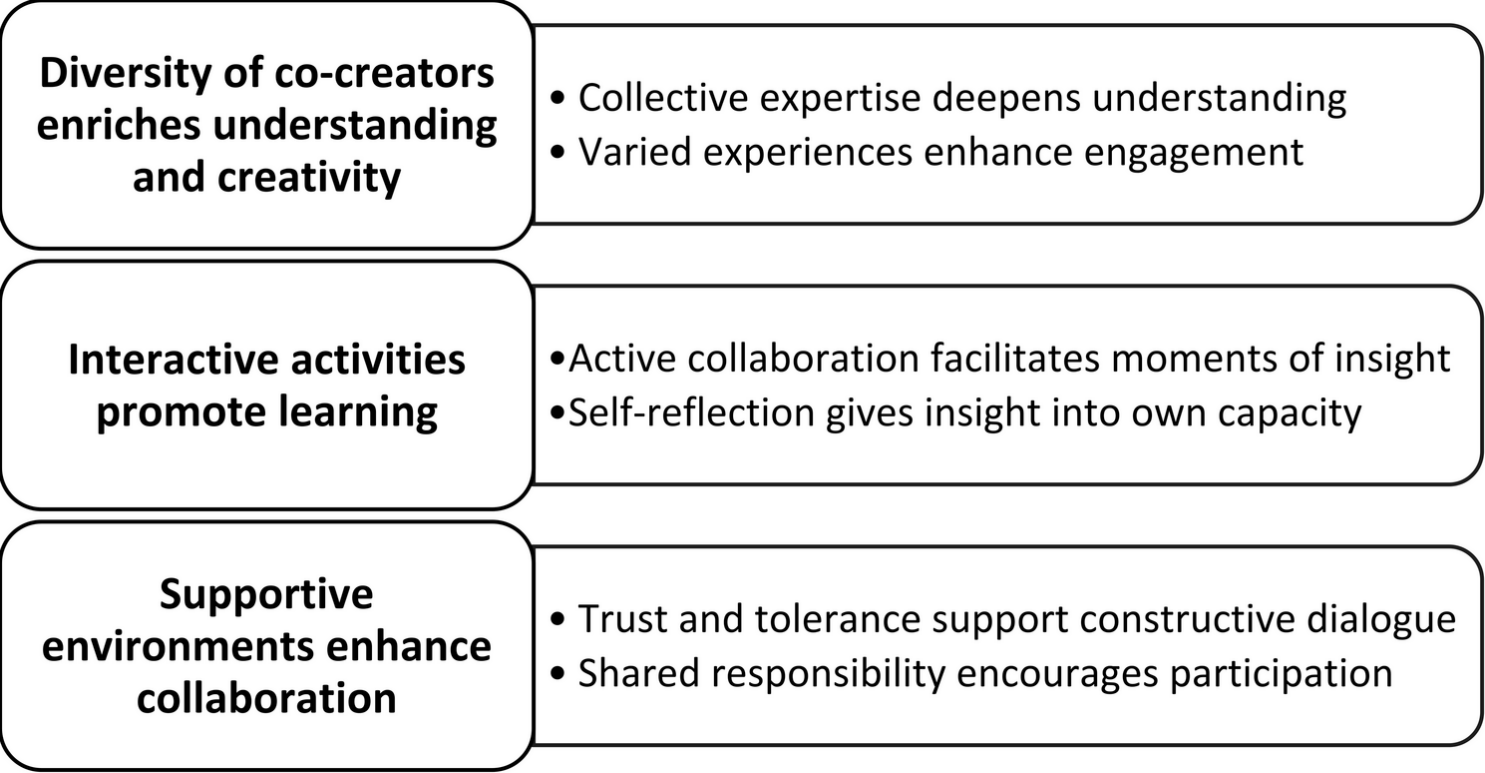
Qualitative content analysis categories and their subcategories

The analysis resulted in three main categories describing the older participants’ experiences of being involved in co-creation: Diversity of co-creators enriches understanding and creativity, Interactive activities promote learning, and Supportive environments enhance collaboration. Six subcategories stem from the main categories (Fig. 1).

### Diversity of co-creators enriches understanding and creativity

This category explores the impact of the diverse backgrounds of the participants on the group discussions. The different backgrounds and expertise gave the older participants new viewpoints and deepened their understanding of the topic. Additionally, the diverse backgrounds stimulated creative discussions in the group, providing new and varied information, which was perceived as valuable and enriching.

#### Collective expertise deepens understanding

The group composition was perceived as an advantage for co-creation. Diverse co-creators, including older persons, researchers, and physiotherapists, enriched the discussion with their unique expertise. As experts in their everyday lives, older persons offered valuable insights into the practical implications of the suggested motor-cognitive exercises while physiotherapists contributed their theoretical and clinical perspectives. Researchers also contributed theoretical knowledge, together with current evidence. This collective expertise provided different points of view, which served as an introduction to the discussions. Moreover, the discussions in the group reinforced the older persons’ understanding of the concept. By doing so, it also affirmed and validated each participants’ contribution.

*“…it’s like this that you have a slightly different background…// [pause]. We had different backgrounds*,* [reflecting back on what the person already said] //. So that’s good. You almost have to have that in a group like this if you want to get something out of it” (Participant 13).*

#### Varied experiences enhance engagement

The diverse group also contributed to open-minded and creative discussions, which were perceived as stimulating, enhancing engagement. The participants noted that one person could initiate the conversation, with other members of the group providing input, propelling the discussion forward.

*“I mean*,* if you have this mixture*,* you reached certain points*,* I imagine*,* when you develop something… where it stops for some*,* but when the knowledge is shared [in the group]*,* and solutions can emerge*,* and then you can take the next step and the next step…yes… I believe that*,* if it is only one experience that solves the problem*,* then it can also get a bit tedious” (Participant 12)*.

When participants shared their past experiences, they became a source for creative brainstorming, generating new ideas. When others acknowledged and built upon their contributions, it reinforced their engagement and commitment to the collaborative effort. Additionally, they highlighted the importance of having the opportunity to contribute and engage, and as one participant expressed: *“we are the ones who understand the problem” (Participant 7)*, while another said it is *“almost criminal not to ask those it concerns[laughter]” (Participant 6).*

### Interactive activities promote learning

This subcategory describes how interactive activities, such as practical testing of motor-cognitive exercises together with others and subsequent discussions, facilitated deeper insights into the concept which accelerated their learning. Additionally, the interactive activities also provided new insights into the participants’ own physical capacity and the importance of exercise in everyday life.

#### Active collaboration facilitates moments of insight

Testing exercises during the workshops triggered new insights into the motor-cognitive concept and its challenge and relevance for balance. One participant described this as:

*P: In the beginning*,* well…this [concept]*,* was misunderstood by many*,* and me too. Like… after the first time it was really unclear*,* what is this good for?*

*I: Exactly. At what stage did it start to become clear*,* or did it clear up?*

*P: It cleared up perfectly when we got to do the practical exercises.*

*I: then the pieces sort of started to fall into place?*

*P: Yes*,* yes. (Participant 7).*

These new insights were supported by active collaboration and reflection together with the other group members. The participants formed novel ideas of how to use and combine motor/physical activities together with a cognitive activity, e.g. walking while talking on the phone or texting.

#### Self-reflection gives insight into own capacity

Together with the knowledge gained from the workshops, self-reflection provided additional value in relation to the older participants’ understanding of their physical abilities, particularly during everyday tasks that require attentional resources, and for some, highlighted the importance of exercise.

*“I had never thought of this*,* what we did*,* all our exercises that the researchers… did with us. I had never really thought of it. It was quite good. Then you had to learn balance*,* and just this thing about working with the brain at the same time. I thought that was great.…” (Participant 13).*

The practical exercise sessions also revealed individual differences in participants’ ability to complete the exercises successfully. Participating in the workshops provided insights into the impact of aging on their physical capacity and participants identified weaknesses in their physical performance that they were not previously aware of. The testing provided a platform for self-reflection, where older participants considered how their physical capabilities affect daily tasks, particularly routine activities.

*“Yes*,* you got us to start thinking in other ways… especially when we got these exercises and then we did them and realized that yes*,* we are probably not that good at this… we walk around at home thinking that we know and can do [meaning importance of balance and exercise in daily life] and then it turns out….you came and brought us down to earth immediately (Participant 8).*

The new awareness of their limitations motivated some to continue to exercise at home after the workshops ended. Some participants also felt empowered and expressed that they would like to take on leadership roles to support peers in this type of exercise. They wanted to motivate and contribute to other people’s well-being in the community.

*“And when I think about it*,* I probably could*,* if it was the case that one really wanted to and if I appreciated it in some way… And thought that*,* that it could contribute… I could get a number of pensioners who don’t do much*,* who could start cycling [more regularly] and exercise a bit more” (Participant 2).*

### Supportive environments enhance collaboration

This subcategory explores participants’ perceptions of collaboration during the workshops. A prerequisite for successful group discussions was a respectful atmosphere, and everyone had the opportunity to express their thoughts. This subcategory also highlights the importance of shared responsibility in taking and giving each other space to contribute.

#### Trust and tolerance support constructive dialogue

The participants reflected on the importance of creating a trusting atmosphere in the group. This could be achieved by allowing sufficient time and space for positive group dynamics to develop.

*“.you always have to start… that you get to know each other a little bit*,* who is in the group*,* that you dare to engage*,* so that was good. It’s good to know other’s names and we got to introduce each other [in pairs]and… I think that was very good” (Participant 7).*

The participants described that trust developed when group members supported and respected each other. This also contributed to feeling seen and heard. The safe and supportive group atmosphere allowed the participants to express their opinions and thoughts openly, without fear of receiving negative feedback or judgmental comments, facilitating constructive discussion. At the same time, participants appreciated individual differences and contributions, which were seen as important and promoted feelings of tolerance and trust. One participant reflected on their own characteristics: “*I am by nature a rather quiet person. But I think that I… No*,* but I thought that…for being me*,* I thought I participated well” (Participant 6).*

#### Shared responsibility encourages participation

Shared responsibility to encourage group participation was seen as a way to empower each member to contribute and ensure a more balanced and inclusive discussion. One participant expressed *“no one was oppressed and everyone’s contribution was appreciated (Participant 7)*, while another participant expressed “*…[you] have to show respect for those who participate in the discussion so that the discussion gets new energy” (Participant 8).* Participants highlighted the importance of a common goal in the group to create room for all group members to share their thoughts. Additionally, participants valued that the discussions had direction, without being too controlled, which also stimulated participation. One participant expressed this as *“You [the researchers] were there and organized things*,* but you didn’t steer us in any way. I mean*,* we got our assignments and we fairly quickly understood what was expected of us” (Participant 6)* while another participant said, *“it wasn’t noticeable that you*,* so to speak*,* steered the conversations in a certain direction*,* so to speak*,* instead you were more there and listened.// and also came up with interesting points of view” (Participant 5).*

Aspects that affected participation and engagement negatively included an imbalance in talking and listening which affected equity in participation. One participant described difficulties in contributing to the discussion when others talked too much *“because when we talked about how to behave and act in a group and it worked really well until the last occasion when I switched group and then it didn’t work and when the others were supposed to talk they… they didn’t listen*,* they talked [only] to each other*,* even if we clearly went through what our [laughter] rules were. I had to raise my voice a bit and assert myself and then I also laid low at times yes…(Participant 1).*

Participants perceived an environment where no one person dominated at the expense of others as a prerequisite for the group to function well. One participant said: *“I think if you sit and talk and discuss*,* things come up more*,* but if someone talks all the time*,* then it [the discussion] gets stifled” (Participant 10).* When space and opportunity were not given, it discouraged participation and resulted in unequal contributions and poorer engagement. Some participants felt that at times the discussions were tangential. In these situations (imbalance and off topic), some participants wished for more governance from the moderators facilitating the discussion. One participant suggested “*maybe you need to steer the discussions a bit more*,* because sometimes they flutter out quite a bit these discussions and one gets into things that are far from the topic at hand” (Participant 1).*

## Discussion

This study explored older persons’ experiences of being involved in co-creation. Participation broadened their knowledge and gave new insights into the subject through interactive workshop activities, such as joint discussions and exercise testing. Participants benefited from others’ experiences, which provided diverse viewpoints and helped contextualize the subject. Fostering an inclusive and permissive atmosphere for discussions and activities was seen as crucial to facilitating participants to contribute in ways that best suit their individual preferences.

Participating in co-creation gave the participants a better understanding of the intervention concept and how it related to everyday life. The interactive workshop activities involved information provided by the researchers, followed by group brainstorming (*Collective expertise deepens understanding*). Participants shared their previous knowledge and experience, including comparing and discussing each other’s contributions (*Diversity of co-creators enriches understanding and creativity)*, which provided new perspectives and enhanced their overall understanding of the concept.

Trying prototype exercises further developed participants’ new knowledge (*Interactive activities promote learning)*. The combination of theoretical knowledge and practice further clarified and deepened their knowledge and understanding of the concept and how it related to everyday life activities. According to Leask [2] prototyping aims to create opportunities for participants to visualize their thoughts and give suggestions for improvement.

The practical sessions where participants tested prototype exercises also provided participants with insights into their own capacity (*Self-reflection gives insight into own capacity)*. This finding is supported by learning theories describing how concrete experiences create meaning [24], arouse reflection on one’s actions, and subsequently result in new learning [25]. For some participants, this experience uncovered previously unrecognized challenges, highlighting the multifaceted benefits of practical sessions within co-creation to promote self-awareness and learning.

Participants developed a sense of social togetherness, mutual learning, and understanding during co-creation. According to the Community of Inquiry Model [25], one important aspect of learning is social presence in the learning situation. Garrison et al. [25] described social presence as the ability of participants to express their individual traits in the group, allowing them to present themselves to others as ’real people.’ Participants also experienced that they could be themselves, discuss, and connect with others in the group, which indicates that they experienced a social presence during the workshops.

The social aspect of co-creation is further fortified in the category *“Supportive environments enhance collaboration.”* When the atmosphere in the group was respectful, it supported participation and engagement by creating possibilities for each participant to express their thoughts, with participants perceiving the atmosphere as positive and inclusive. This aligns with a previous study where the importance of building relations and social connections impacted group inclusivity [26]. PAAR highlights the importance of creating an appreciative, trustful, and open-minded climate in the co-creation group [27]. Additionally, participants highlighted the importance of shared responsibility in creating a permissive group atmosphere. This reflects the concept of ownership in co-creation. According to Leask [2], each co-creator takes responsibility for investing in the process and, by that, supports a sense of belonging for all group members, creating ownership. In contrast, a negative atmosphere is a hindrance to inclusive discussion. An imbalance between talking and listening was perceived as the greatest threat to participation. According to Collins [28], to achieve functional cooperation in a group, each member needs to be responsive to other team members’ emotional states, as well as be able to balance their own emotions in relation to the rest of the group.

Workshops can be valuable for co-creation, but poor group dynamics can hinder the process. In our study, participants noted that they occasionally desired more guidance from the moderators during discussions. However, increased governance can disrupt the balance of power within the group and reduce the level of ownership experienced by the target population participants. In such cases, the moderator must carefully balance providing direction and maintaining participant ownership. Ya-Hui et al. recommends clearly establishing group norms and rules at the outset to positively influence group behavior subsequently resulting in a more satisfying experience for all members [29]. Even though the workshops included discussions about how to interact in a respectful way, sometimes these agreements were overstepped.

### Methodological discussion

The methods used in this study, qualitative interviews, are key to exploring end-users’ experiences of research involvement– in this case, co-creation workshops. Participants of the co-creation study (*n* = 17) were invited to be interviewed at the last workshop and 76% (*n* = 13) agreed to be interviewed and participated in this sub-study. The high participation rate ensured that we captured a broad range of older participants’ experiences. However, there is a chance that those who did not participate had a more negative experience, which may bias the findings. More males (*n* = 9) than females (*n* = 4) participated in the interviews (and in the co-creation study). This contrasts with previous fall prevention research indicating that, overall, more women than men participate in this field of research [8]. There may be differences in how women and men experience co-creation, and this has not been accounted for, or explored in our study.

Data collection was conducted using semi-structured interviews with an interview guide. A fundamental limitation of semi-structured interviews is the use of predetermined questions [29], which may lead participants to discuss only the topics mentioned in the interview questions. To avoid this, the researcher strove to ask questions as openly as possible and encouraged the participants to elaborate on their experiences, using probing techniques. Conversely, according to Wilson [30] a predetermined interview guide ensures that the same topics are addressed in each interview, which leads to more coherent data.

The participants in this study had previously participated in workshops to develop a fall prevention intervention, and this study serves as a continuation of those workshops by exploring their experiences with co-creation research. Thus, while the participants were co-creators of the intervention, they were informants in this study but not collaborators on the co-creation research project, nor this study, which is a limitation of the project. However, the research team undertook member checking in each workshop, to help ensure they were appropriately interpreting the participants input. Nevertheless, participants’ insights at the analysis and manuscript drafting stages could have provided new perspectives and validation of the study findings.

This study’s authors have previous experiences in aging and health, which have influenced data collection, analysis, and interpretation of findings. To avoid researcher bias, the author group met regularly to discuss the analysis and reflect on their own experiences and potential biases which could affect the findings. By reflecting openly on these and acknowledging the influences, it ensures transparency in the study. The interviews were conducted by the first author, a researcher who functioned as a moderator in the workshops, which could have created bias. However, participating in the workshops gave the interviewer an insider perspective, making it easier for the interviewer to ask relevant follow-up questions when needed.

### Implication for practice

When developing co-creation processes involving groups of individuals, it is important to provide time and space for a group community to develop in a safe atmosphere. This requires time to be spent on the initial phase of the process so that participants feel that their participation is valued and that each participant is seen and heard. This confirms that the participants’ contributions are valued and their competence in contributing. The group community also supports peer learning and understanding, which facilitates intervention development.

## Conclusions

Participating in co-creation with other co-creators gave older persons valuable experiences, expanding their subject knowledge and giving them more profound insights into their abilities in daily life. Combining different interactive activities in the workshops enriched their learning experience. Inclusion and trust within the group were imperative for effective participation and engagement, providing everyone with opportunities to express their thoughts and ideas. For this to happen, time and space must be provided at the start of the process for a positive group dynamic to develop.

## Electronic supplementary material

Below is the link to the electronic supplementary material.

Supplementary Material 1

## Data Availability

No datasets were generated or analysed during the current study.
