# Supplementary material for: In their own words: older persons’ experiences of participating in co-creation
Source: Res Involv Engagem. 2025 May 26;11:56. doi: 10.1186/s40900-025-00725-z (PMC12105406; doi:10.1186/s40900-025-00725-z)
Supplement: Supplementary file 1 — Supplementary Material 1 [file 40900_2025_725_MOESM1_ESM.pdf]

Supplementary Material

Supplementary Table 1. *GRIPP2 Short Form for the current study.*

Section and topic	Item	Reported on page No
1: Aim	Report the aim of PPI in the study	4
2: Methods	Provide a clear description of the methods used for PPI in the study	4-6
3: Study results	Outcomes – Report the results of PPI in the study, including both positive and negative outcomes.	7-13, Figure 1
4: Discussion and conclusions	Outcomes – Comment on the extent to which PPI influenced the study overall. Describe positive and negative effects.	13-16
5: Reflection/critical perspective	Comments critically on the study, reflecting on the things that went well and those that did not, so others can learn from this experience.	16-17
